# Radiation‐Enhanced AF1q Moves Center Stage as a Key Driver to Favorable Tumor Stage in Rectal Cancer Patients

**DOI:** 10.1002/cam4.70658

**Published:** 2025-03-10

**Authors:** Elisabeth S. Gruber, Georg Oberhuber, Elisabeth Gurnhofer, Robert Eferl, Gerald Timelthaler, Béla Teleky, D. I. Dietmar Georg, Joachim Widder, William Tse, Lukas Kenner

**Affiliations:** ^1^ Division of Visceral Surgery, Department of General Surgery Medical University Vienna Vienna Austria; ^2^ Department of Experimental and Animal Pathology Clinical Institute of Pathology, Medical University Vienna Vienna Austria; ^3^ PIZ—Patho Im Zentrum GmbH, St. Poelten Lower Austria Austria; ^4^ Center for Cancer Research Medical University Vienna Vienna Austria; ^5^ Comprehensive Cancer Center Medical University Vienna Vienna Austria; ^6^ Center for Biomedical Research and Translational Surgery Medical University Vienna Vienna Austria; ^7^ Department of Radiation Oncology Medical University of Vienna Vienna Austria; ^8^ Department of Medicine, School of Medicine Case Western Reserve University Cleveland Ohio USA; ^9^ Immune Oncology Program, Case Comprehensive Cancer Center Case Western Reserve University Cleveland Ohio USA; ^10^ Unit of Laboratory Animal Pathology University of Veterinary Medicine Vienna Vienna Austria; ^11^ Christian Doppler Laboratory for Applied Metabolomics Medical University Vienna Vienna Austria; ^12^ Center for Biomarker Research in Medicine (CBmed) Graz Styria Austria

## Abstract

**Background:**

Enhanced protein expression of ALL1‐fused gene from chromosome 1q (*AF1Q*) after (chemo)radiotherapy has been described in vitro, but is largely understudied in gastrointestinal cancer. We aimed to investigate AF1q expression in rectal cancer (RC) patients treated with short‐term radiation therapy and a possible correlation with markers crucial for RC prognosis.

**Methods:**

A cohort of 75 RC patients scheduled for surgery was defined and patients with moderately locally advanced tumors (cT3Nx) received preoperative hyperfractionated short‐term radiation therapy (cumulative dose 25 Gy). Immunohistochemical analysis was conducted to assess AF1q, STAT1, IDO1 and other prognostic markers (CD3/CD8—Immunoscore, PD‐L1) and marker correlations were evaluated.

**Results:**

Irradiated tumors exhibited significantly higher AF1q expression than treatment‐naïve samples (*n* = 60: AF1q + to AF1q+++ 98.3% (*n* = 59), AF1q‐ 1.7% (*n* = 1) vs. *n* = 15: AF1q + 78.6% (*n* = 11), AF1q‐ 21.4% (*n* = 4); *p* < 0.001). Specifically, irradiated tumors showed high STAT1, but low IDO1 expression compared to treatment‐naïve samples (*p* = 0.019 and *p* = 0.015, respectively). Overall, enhanced tumoral AF1q expression was associated with negative lymph node stage (*p* = 0.012) as well as with diminished expression of STAT1 (*r*_s_ = −0.468, *p* = 0.038) and IDO1 (*r*_s_ = −0.246, *p* = 0.020).

**Conclusion:**

AF1q is expressed in RC, especially after short‐term radiation therapy. Here, AF1q may support tumor suppression, possibly through the involvement of the pro‐apoptotic STAT1 axis. Further mechanistic evidence and investigation involving a larger patient cohort are needed to validate a radiation‐induced, AF1q‐driven tumor‐suppressing effect, which may impact RC patient outcomes.

## Graphical abstract

Short‐term radiation therapy induces oncogenic AF1q expression in rectal cancer that correlates with negative lymph node stage AF1q expression associates with high STAT1 and low IDO1 expression in irradiated tumor samples The favorable effects of short‐term radiation therapy on tumor biology might be enhanced by an AF1q‐driven pro‐apoptotic signaling axis AF1q may be a promising therapeutic target responsive to radiation therapy.

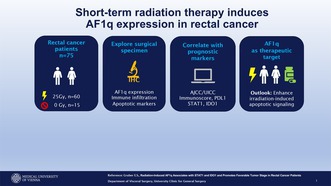

## 1 Introduction

ALL1‐fused gene from chromosome 1q (*AF1Q*) was previously identified as a negative prognostic marker in gastrointestinal cancer [1, 2] as well as various other solid malignancies [3, 4, 5, 6, 7, 8, 9] and exhibits oncogenic potential by modulating multiple signaling pathways [1, 2]. *AF1Q* was first described as a *MLL* fusion partner in acute myeloid leukemia patients with a t(1; 11)(q21; q23) translocation (*MLLT11*) [10] and since then, reports indicate that it is involved in the entire spectrum of oncogenic processes, from tumor initiation to dissemination—the latter has previously been demonstrated in colorectal cancer (CRC) patients [2]. Of note, AF1q unfolds its potential by mediating downstream transcription factors, such as NF‐κB known to regulate BCL2‐associated agonist of cell death (BAD), a protein involved in pro‐ as well as anti‐apoptotic signaling cascades [4]. In squamous carcinoma cells, AF1q has been demonstrated to enhance irradiation‐induced apoptosis and the same effect has been observed with doxorubicin‐induced apoptosis also in hepatocellular carcinoma cells [4, 5]. Transcription factor signal transducer and activator of transcription 1 (STAT1) is recognized as a good prognostic factor in CRC known for its role in tumor immunosurveillance [11, 12, 13] as well as apoptotic signaling [4, 5, 14, 15], but its association with AF1q remains unexplored.

Locally advanced rectal cancer (RC) is treated with preoperative (chemo)radiotherapy to reduce tumor size and prevent local recurrence [16]. However, the effects of radiation on immune cell and immune checkpoint marker expression in RC have yielded conflicting results. We previously found a significant decrease in CD3 and CD8 positive T cells in samples of RC patients treated with preoperative hyperfractionated short‐term radiation therapy [17]. Several other studies resulted in controversial results with regard to immune cell and immune checkpoint marker expression, which is most likely attributable to the different preoperative protocols investigated [18, 19, 20, 21, 22, 23, 24, 25]. As for clinical applicability, PD‐L1 has shown biomarker potential in mismatch‐repair deficient CRC [26, 27], while the Immunoscore, which represents the prognostic tumor immune cell contexture in (C)RC, is being translated into clinical practice [18, 28]. The enzyme Indoleamine 2,3‐dioxygenase 1 (IDO1) is regulated by the interferon‐driven STAT1 axis and contributes to the balance of the tumor microenvironment (TME) [29, 30, 31]. Aberrant IDO1 expression is involved in tumor immune escape, which makes it an anti‐cancer target under investigation. In animal experiments, STAT1‐dependent IDO1 expression has been demonstrated on intestinal Paneth cells that corresponded to cells in human CRC [12]. IDO1 blockade has shown promise in suppressing CD8 T cell apoptosis in the TME of CRC and sensitizing tumor cells to radiation‐induced cell death [32, 33]. IDO1 expression is upregulated in response to (radio)chemotherapy in patients with esophageal squamous cell cancer and predicts poor response and prognosis [34]. The effects of IDO blockade seem to depend on (radio‐)chemotherapy as demonstrated in mouse models of glioblastoma and melanoma [35, 36].

Based on this knowledge, we here aimed to investigate whether AF1q expression is particularly enhanced in RC after hyperfractionated short‐term radiation therapy and evaluate the possible impact on patient outcome including tumor stage and postoperative complications. Additionally, we investigate the potential associations between AF1q and STAT1 expression, CD3/CD8 density, the Immunoscore as well as PD‐L1 and IDO1 expression.

## 2 Results

**FIGURE 1:**
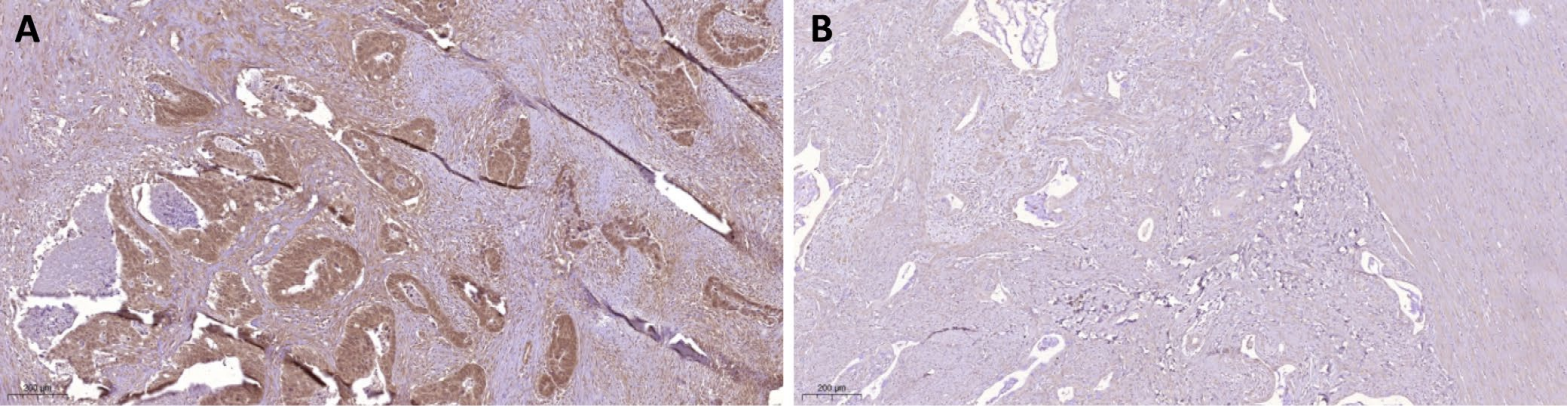
AF1q expression in irradiated (A) vs. treatment‐naïve (B) RC samples. (A) High AF1q expression following hyperfractionated short‐term radiation therapy with a cumulative dose of 25 Gy vs. (B) low AF1q expression following upfront surgery.

**FIGURE 2:**
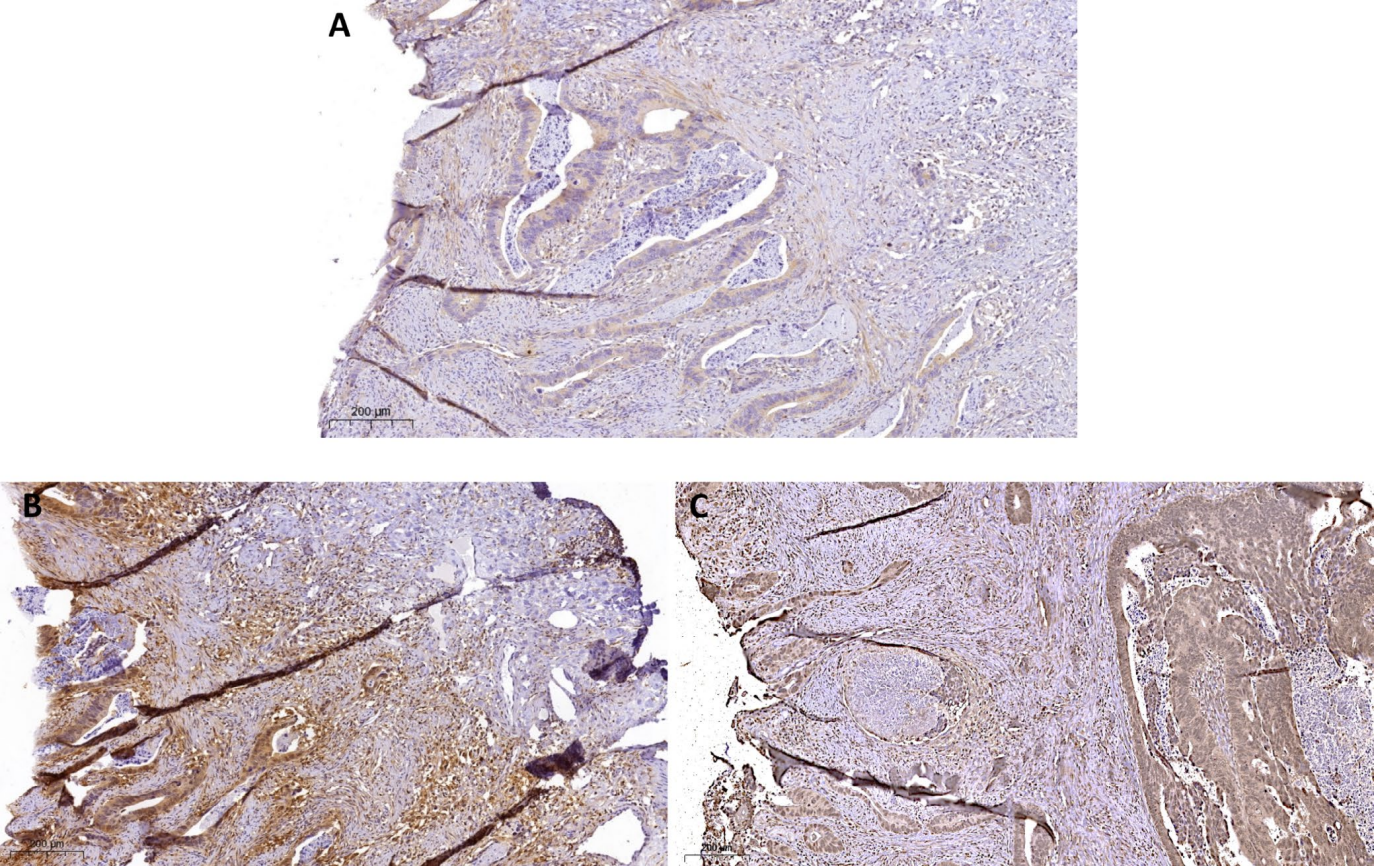
AF1q (A), STAT1 (B) and IDO1 (C) expression in corresponding irradiated RC samples. (A) High AF1q expression corresponds with (B) high STAT1 and (C) low IDO1 expression following hyperfractionated short‐term radiation therapy with a cumulative dose of 25 Gy.

Differences were observed between irradiated and treatment‐naïve tumors, with significantly enhanced AF1q expression in irradiated tumor samples (*p* < 0.001). Among the 60 irradiated tumors, 32 (54.2%) exhibited low AF1q expression, 20 (33.9%) showed moderate, and 7 (10.2%) showed high AF1q expression. Only 1 tumor (1.7%) displayed no AF1q expression. In the group of 15 treatment‐naïve tumors, 11 (78.6%) showed low AF1q expression, whilst 4 (21.4%) tumors showed no expression. None of the treatment‐naïve tumors showed moderate or high AF1q expression. AF1q was also found to correlate with negative lymph node stage (*p* = 0.012), but no significant correlations were observed with other patient and tumor characteristics (Table 1). Regarding STAT1 and immune markers, we observed an inverse correlation between AF1q expression and the expression of STAT1 (*r*_s_ = −0.468, *p* = 0.038) and IDO1 (*r*_s_ = −0.246, *p* = 0.020). However, no correlation was found between STAT1 and IDO1 expression. Irradiated samples exhibited high STAT1 expression but low IDO1 expression compared to treatment‐naïve samples (*p* = 0.019 and *p* = 0.015, respectively). Marker expression in irradiated and treatment‐naïve samples is shown in Figures 1 and 2, respectively. No significant correlations were observed with other RC prognostic markers, including the number of T cells (CD3, CD8), the Immunoscore, or PD‐L1 expression (Table 2).

**TABLE 1:** Patient and tumor characteristics in relation to AF1q expression.

Factors		AF1q^+^			AF1q^−^	*p*
		Low	Moderate	High		
Study cohort	75 (100)	43 (57.3)	19 (25.3)	6 (8.1)	7 (9.3)	
Sex						n.s.
Male	44 (58.7)	26 (34.7)	9 (12.0)	5 (6.7)	4 (5.3)	
Female	31 (41.3)	17 (22.7)	10 (13.3)	1 (1.3)	3 (4.0)	
Age	77 (51–94)					n.s.
< 77 years	34 (45.3)	15 (20.0)	11 (14.7)	4 (5.4)	4 (5.3)	
≥ 77 years	41 (54.7)	28 (37.3)	8 (10.7)	2 (2.7)	3 (4.0)	
Neoadjuvant RT						**< 0.001**
Yes	60 (80.0)	32 (42.7)	19 (25.3)	6 (8.0)	3 (4.0)	
No	15 (20.0)	11 (14.7)	0 (0.0)	0 (0.0)	4 (5.3)	
AJCC/UICC						**0.012**
UICC< III	46 (61.3)	29 (38.7)	12 (16.0)	3 (4.0)	2 (2.6)	
UICC≥ III	29 (38.7)	14 (18.7)	7 (9.3)	3 (4.0)	5 (6.7)	
Tumor grade		43 (57.3)	19 (25.3)	6 (8.0)	7 (9.3)	n.s.
Good (G1)	1 (1.3)	0 (0.0)	1 (1.3)	0 (0.0)	0 (0.0)	
Moderate (G2)	63 (84.0)	36 (48.0)	15 (20.0)	1 (1.3)	7 (9.3)	
Poor (G3)	11 (14.7)	7 (9.3)	3 (4.0)	1 (1.3)	0 (0.0)	
Postoperative complications (CD≥ III)						n.s.
Yes	8 (10.7)	7 (9.3)	0 (0.0)	0 (0.0)	1 (1.4)	
No	67 (89.3)	36 (48.0)	19 (25.3)	6 (8.0)	6 (8.0)	
Local recurrence						n.s.
Yes	3 (4.0)	3 (4.0)	0 (0.0)	0 (0.0)	0 (0.0)	
No	72 (96.0)	40 (53.3)	19 (25.3)	6 (8.0)	7 (9.4)	
Progressive disease						n.s.
Yes	15 (20.0)	8 (10.6)	2 (2.7)	2 (2.7)	3 (4.0)	
No	60 (80.0)	35 (46.7)	17 (22.7)	4 (5.3)	4 (5.3)	

*Note:* Numerical/ordinal variables are described as numbers (percentages), continuous variables as median (standard deviation); RT – radiotherapy (25Gy), CD – Clavien‐Dindo classification for postoperative complications. Significant values are in bold.

**TABLE 2:** STAT1 and immune marker expression in relation to AF1q expression.

Markers	*n* (%)	−	+	Marker^+^		*p*	
				AF1q^+^	AF1q^−^		
STAT1	75 (100)	29 (38.0)	46 (62.0)	15 (33.0)	31 (67.0)	***r***_**s**_ **= −0.468 (*p =* 0.038)**	n.s.
IDO1	75 (100)	24 (32.0)	51 (68.0)	5 (8.8)	46 (90.2)	***r***_**s**_ **= −0.246 (*p =* 0.020)**	
CD3	75 (100)	4 (5.3)	71 (94.7)	65 (91.5)	6 (8.5)	n.s.	
CD8	75 (100)	6 (8.0)	69 (92.0)	63 (91.3)	6 (8.7)	n.s.	
Immunoscore	75 (100)	26 (34.7)	45 (60.0)	41 (68.3)	8 (31.7)	n.s.	
PD‐L1	75 (100)	37 (49.0)	38 (51.0)	34 (91.9)	4 (0.1)	n.s.	

*Note:* Numerical/ordinal variables are described as numbers (percentages). For concise data presentation, marker expression here is summarized as positive (low, moderate, high) versus negative. Significant values are in bold.

## 3 Discussion

AF1Q, a gene implicated in various malignancies, has been associated with poor outcomes in gastrointestinal cancers [1, 2]. In general, AF1q can interfere with key oncogenic pathways and enhance tumor suppressor pathways, promoting a shift towards less aggressive tumor phenotypes; in addition, it may influence the expression of genes involved in DNA repair, apoptosis, and cell cycle regulation, enhancing the sensitivity of tumor cells to radiation‐induced damage [4, 5, 9]. With regard to CRC, there is mechanistic evidence suggesting that AF1q plays a role in tumor development and progression [2]. In the context of esophageal cancer, our research has demonstrated an association of AF1q with markers of the oncogenic STAT pathway [1], which is involved in cell growth and immune function in (C)RC [37]. In this study, we aimed at identifying AF1q as an oncogenic driver in RC, potentially amplified by short‐term radiation therapy. We also sought to evaluate its role in driving the STAT1 pathway and IDO1 expression, both of which are crucially involved in CRC regulation. We found AF1q to be overexpressed in RC samples through short‐term radiation therapy. These samples concurrently exhibited high STAT1 but low IDO1 expression. However, no correlation was found between AF1q and key biomarkers for RC prognosis, such as CD3, CD8, the Immunoscore, and PD‐L1.

AF1q's role as a multi‐faceted oncogene mediating signaling pathways crucial for hematologic and solid malignancies has been well‐documented in selected studies [1, 4, 5, 7, 8, 9, 10, 38, 39, 40]. Interestingly, AF1q has been reported to drive aberrant BAD expression, enhancing irradiation‐induced apoptotic effects in human squamous cancer cells after a single dose of 10 Gy [4]. Our study confirmed increased expression of AF1q in RC samples treated with 25 Gy of hyperfractionated short‐term radiation therapy compared to treatment‐naïve samples.

Radiation therapy is known to exhibit profound effects on the interferon‐axis and immune cell infiltration within the tumor microenvironment; especially, (1) the expression of type I and II interferons, leading to the activation of downstream immune effector pathways that bolster anti‐tumor immunity and (2) enhancing the infiltration of immune cells, such as T cells, into the tumor microenvironment post‐radiation to improve immune‐mediated tumor destruction [18, 19, 24, 25, 32, 41]. Specifically, radiation therapy has been reported to trigger pro‐immunogenic effects, especially after low doses (2–15 Gy) [41]. The patient samples analyzed in our study were selectively treated with short‐term radiation therapy, which uses a lower dose regime compared to conventional radio(chemo)therapy (cumulative dose 25 Gy vs. 50 Gy). In addition, this protocol introduces differences in fractionation and sequencing that could potentially influence the TME, steering it towards an anti‐tumor response and enhancing immune checkpoint expression [21, 41]. However, in our recent study, we observed a reduction in CD3 and CD8 T cells in RC samples following short‐term radiation therapy [17]. Given our findings that AF1q is predominantly increased in irradiated samples and is associated with STAT1, a key player in immunosurveillance, we hypothesize that this AF1q‐STAT1 axis could potentially stimulate T cell response and immune checkpoint expression. Ultimately, we found no evidence of enhanced T cell density nor immune checkpoint expression in association with AF1q expression. Firstly, the robust yet inverse relationship between STAT1 and AF1q expression suggests that in a TME with high AF1q expression, STAT1 appears to be actively suppressed, potentially to inhibit a tumor‐promoting effect that is dependent on phosphorylation. As a result, AF1q is associated with low levels of IDO1 expression, which is dependent on STAT1. However, our subgroup analysis revealed an increase in STAT1 expression following radiation therapy, while IDO1 expression remained low. As demonstrated in previous studies, a dose of 25 Gy of photon‐irradiation led to a significant reduction in the density of T cells within the TME of RC [17]. This implies that the interferon‐axis, which is known for enhancing inflammatory immune cell infiltration, may be diminished in tumors treated after this specific short‐term radiation protocol [42]. It suggests that irradiation could primarily drive STAT1 phosphorylation towards a pro‐apoptotic axis, promoting tumor shrinkage, potentially leading to the decoupling of IDO1 expression from STAT1‐dependency by either altering the signaling dynamics within the tumor microenvironment, leading to changes in the regulatory control of IDO1 expression or by alternate STAT1‐independent pathways, such as direct DNA damage response mechanisms or other transcription factors activated by radiation. Suppressed IDO1 activity in the tumor microenvironment might be directly inhibited by AF1q at the transcriptional level or by enhancing anti‐tumor immunity and reducing immune suppression. IDO1 expression is often associated with tumor immune escape. Therefore, the observed low levels of tumoral IDO1 support our findings of a reduced immunosuppressive TME following 25 Gy of photon‐irradiation. Furthermore, previous research has shown that suppressed IDO1 activity can lead to increased apoptosis of CD8 T cells in the TME of a CRC mouse model and make CRC cells more susceptible to irradiation‐induced cell death [33]. In line with these findings, we observed an overexpression of AF1q, induced by irradiation, in patients with a low histopathological tumor stage. However, these findings contrast with our previous research on esophageal cancer patients, where we identified AF1q as a predictor of poor prognosis but also with enhanced AF1q expression after neoadjuvant therapy [1]. A study on esophageal squamous cell cancer revealed similar results with respect to IDO1 enhanced after neoadjuvant therapy and associated with poor pathological response and prognosis [34]. This discrepancy suggests that different tumor entities and neoadjuvant treatment protocols used may yield divergent results, warranting further investigation.

In conclusion, our study suggests that 25 Gy of hyperfractionated accelerated short‐term radiation therapy induces aberrant AF1q expression that may support tumor shrinkage and leads to a favorable outcome in patients with RC. Our findings indicate that short‐term radiation therapy in particular may activate the pro‐apoptotic STAT1 axis while inhibiting IDO1‐driven immune escape, both associated with AF1q overexpression. Of note, potential selection bias in discerning a favorable outcome through radiation therapy with AF1q expression or through radiation therapy alone must be ruled out by investigating larger patient cohorts. This potential selection bias limits the generalizability of our findings. Finally, the retrospective design of the study inherently limits the ability to draw causal inferences. Prospective studies are needed to substantiate our findings.

Collectively, our findings underscore the crucial influence of AF1q in gastrointestinal oncogenesis that can be driven by photon‐irradiation to modulate oncogenic signaling towards a favorable tumor biology. This further suggests that AF1q may be a promising therapeutic target responsive to radiation therapy in terms of enhancing the efficacy of existing treatments and potentially leading to the development of novel therapeutic strategies aimed at boosting its expression or mimicking its activity in tumors. As a predictive biomarker, AF1q could serve to identify patients who are more likely to respond favorably to radiation therapy, allowing for more personalized treatment approaches, such as more tailored and effective treatment plans. Consequently, AF1q expression may serve to guide clinical decision‐making.

## 4 Materials/Subjects and Methods

### 4.1 Patient Cohort and Selection of HE‐Stained Slides

As for patient cohort selection, inclusion criteria comprised patients aged 18 years and older, diagnosed with locally advanced RC that qualified for a hyperfractionated accelerated short‐term preoperative radiation therapy protocol [42]. Exclusion criteria included patients with incomplete medical records, those who received prior treatment for RC outside the study parameters, and patients with concomitant serious illnesses that could confound results.

Clinical tumor staging was classified according to the respective AJCC/UICC staging system (TNM classification) for RC [43]. Diagnosis was verified by tumor biopsy determining the specific tumor subtype and tumor grade. Final histology was collected from pathological reports which enclosed a standardized, detailed description of tumor histology, subtypes, and morphological features as well as the final WHO tumor grade and AJCC/UICC tumor stage [16].

In total, we retrospectively analyzed 215 cases operated on for RC between 2000 and 2009 at the Medical University of Vienna, Austria. 60 patients with moderately locally advanced RC who had fulfilled inclusion criteria to be treated within a hyperfractionated accelerated short‐term preoperative radiation therapy protocol [42] were randomly selected. In short, patients with primary resectable RC at MRI‐based clinical stage T3Nx who were assessed as being at increased risk of local recurrence received 25 Gy within 1 week (Monday to Friday, 2.5 Gy given twice daily with 6 h interval). Surgery took place the following week.

The control group was composed of patients who did not receive a hyperfractionated accelerated short‐term preoperative radiation therapy protocol due to clinical contraindications, patient preference, or logistical factors; these patients were otherwise managed according to standard clinical protocols and were matched to the study group based on age, sex, and disease stage. The treatment protocol for the control group included primary resection for RC. Patient characteristics such as baseline demographics, comorbidities, and clinical features were documented to ensure comparability.

In total, 15 patients with RC undergoing primary resection were selected as a control group. According to oncologic consensus guidelines, partial mesorectal excision for tumors located in the upper third and total mesorectal excision for tumors located in the middle and lower rectum with a stapled anastomosis were performed; protective ileostomies were added at the surgeon's discretion. HE‐stained histopathological tumor samples were examined together with a board‐certified pathologist; here, at least 5 HE slides were examined per case and selected for immunohistochemical processing. Good Scientific Practice (GSP) Guidelines were used handling patient data. Analyzation of radiographic data was made in accordance with the response evaluation criteria in solid tumors (RECIST). Approval of the study was obtained by the Medical University of Vienna's Ethics Committee (EC #1197/2019).

### 4.2 Immunohistochemical Procedures and Reagents

As for tissue preparation, paraffin‐embedded tissue sections were cut at 1 μm thickness and placed on glass slides. Regarding the staining protocol, slides were deparaffinized in xylene, rehydrated through graded alcohols, and subjected to antigen retrieval using a Bond Epitope Retrieval 1 solution (Leica Biosystems Inc., Buffalo Groove, IL; Cat no. AR9961), blocking of unspecific binding sites was performed using a 2% goat serum. We used the following primary monoclonal antibodies according to established staining protocols: anti‐AF1q (Abcam, ab109016, 1:200), anti‐IDO1 (Biolegend, San Diego, California/USA; Cat no. 122402, 1:80,), anti‐STAT1 (Santa Cruz, Dellas, Texas/USA; Cat no. sc‐592, 1:500), anti‐CD3 (Abcam, Cambridge, Cambridgeshire/UK; Cat no. ab5690) 1:300, anti‐CD8 (Abcam, Cambridge, Cambridgeshire/UK; Cat no. ab4055; 1:300), anti‐PD1 (Abcam, Cambridge, Cambridgeshire/UK; Cat no. ab137132) 1:75, anti‐PD‐L1 (Abcam, Cambridge, Cambridgeshire/UK; Cat no. ab205921; 1:100).

Staining was performed using a Leica Bond RX Automated Stainer (Leica Products/Equipment, Leica Microsystems Inc., Buffalo Groove, IL). Slides were incubated for 30 min at 95°C and dewaxed with Leica Bond Dewax solution (Leica Biosystems Inc., Buffalo Groove, IL; Cat no. AR9222). The Leica Bond Refine Detection kit (Leica Biosystems Inc., Buffalo Groove, IL; Cat no. DS9800) was used for the visualization of primary antibody binding with diaminobenzidine chromogen and a hematoxylin counterstain. Primary antibodies were diluted in Leica Bond Antibody Diluent buffer (Leica Biosystems Inc., Buffalo Groove, IL; Cat no. AR9352).

### 4.3 Evaluation and Scoring of Immunohistochemical Results

Analogue quantification of protein expression was performed by two independent, experienced board‐certified pathologists in a double‐blinded manner. Inter‐rater reliability was assessed using Cohen's kappa coefficient, which yielded a value of 0.85, indicating an excellent level of agreement.

Marker density was scored based on the percentage of positively stained cells and the intensity of staining. A semi‐quantitative scale (e.g., 0–3 for intensity: negative (0) or positive in terms of a low (1), moderate (2) or high (3) expression) was applied. A specimen was considered positive when at least 50% of tumor or immune cells showed moderate or strong cytoplasmic (AF1q, STAT1, IDO1) or membranous (CD3, CD8, PD1, PD‐L1) marker expression on the whole slide, respectively. If different results were obtained by the two independent investigators, samples were re‐evaluated together, and an agreed final score was determined.

### 4.4 Statistical Analysis

As for descriptive statistics, means, medians, standard deviations, and ranges were calculated for continuous variables, while frequencies and percentages were determined for categorical variables. Comparisons between tumor marker expression and patient and tumor characteristics were performed using the *χ* ^2^ test, Wilcoxon rank sum test, and Spearman's rank correlation coefficient as appropriate. As for Table 2, data are depicted as frequencies/percentages of positive/negative STAT1/IDO1 marker expression in relation to AF1q expression (summarized as positive (low, moderate, high) vs. negative for concise data presentation—as described in the “note” section); for statistical analysis of significance, the ordinally scaled groups (low, moderate, high vs. negative) were used and tested using the Spearman rank correlation coefficient (non‐parametric test, since data does not have to be normally distributed and the variables only have to be ordinal scaled; a rank correlation can also be calculated for small samples and outliers).

Data were analyzed using IBM SPSS Statistics Version 28 software. A two‐sided *p‐*value of less than 0.05 was considered statistically significant.

## Author Contributions

**Elisabeth S. Gruber:** conceptualization (lead), data curation (lead), formal analysis (lead), funding acquisition (lead), investigation (lead), methodology (lead), project administration (lead), resources (equal), validation (supporting), visualization (lead), writing – original draft (lead), writing – review and editing (supporting). **Georg Oberhuber:** conceptualization (equal), data curation (equal), formal analysis (supporting), investigation (lead), methodology (supporting), resources (equal), supervision (lead), writing – review and editing (equal). **Elisabeth Gurnhofer:** investigation (supporting), methodology (supporting), resources (supporting). **Robert Eferl:** conceptualization (supporting), investigation (supporting), methodology (supporting), resources (supporting), supervision (supporting), validation (supporting), writing – review and editing (supporting). **Gerald Timelthaler:** resources (supporting). **Béla Teleky:** conceptualization (supporting), resources (lead), supervision (supporting), validation (supporting), writing – review and editing (supporting). **D. I. Dietmar Georg:** conceptualization (supporting), investigation (supporting), resources (lead), supervision (supporting), validation (lead), writing – review and editing (lead). **Joachim Widder:** conceptualization (supporting), investigation (supporting), resources (lead), supervision (supporting), validation (lead), writing – review and editing (lead). **William Tse:** conceptualization (supporting), investigation (supporting), supervision (lead), validation (lead), writing – review and editing (lead). **Lukas Kenner:** conceptualization (supporting), investigation (supporting), resources (supporting), supervision (lead), validation (lead), writing – review and editing (lead).

## Ethics Statement

Approval of the research protocol by an Institutional Reviewer Board: Medical University of Vienna's Ethics Committee (EC #1197/2019).

## Consent

The authors have nothing to report.

## Conflicts of Interest

The authors declare no conflicts of interest.

## Data Availability

Data available on request due to privacy/ethical restrictions: The data that support the findings of this study are available on request from the corresponding author. The data are not publicly available due to privacy or ethical restrictions.
